# Visible and near-infrared spectroscopy and deep learning application for the qualitative and quantitative investigation of nitrogen status in cotton leaves

**DOI:** 10.3389/fpls.2022.1080745

**Published:** 2022-12-20

**Authors:** Qinlin Xiao, Na Wu, Wentan Tang, Chu Zhang, Lei Feng, Lei Zhou, Jianxun Shen, Ze Zhang, Pan Gao, Yong He

**Affiliations:** ^1^College of Biosystems Engineering and Food Science, Zhejiang University, Hangzhou, China; ^2^Key Laboratory of Spectroscopy Sensing, Ministry of Agriculture and Rural Affairs, Hangzhou, China; ^3^School of Information and Electronic Engineering, Zhejiang University of Science and Technology, Huzhou, China; ^4^School of Information Engineering, Huzhou University, Huzhou, China; ^5^College of Mechanical and Electronic Engineering, Nanjing Forestry University, Nanjing, China; ^6^Hangzhou Raw Seed Growing Farm, Hangzhou, China; ^7^Key Laboratory of Oasis Eco-Agriculture, College of Agriculture, Shihezi University, Shihezi, China; ^8^College of Information Science and Technology, Shihezi University, Shihezi, China

**Keywords:** cotton, leaf nitrogen content, spectra, deep learning, visible and near-infrared spectroscopy (Vis-NIR)

## Abstract

Leaf nitrogen concentration (LNC) is a critical indicator of crop nutrient status. In this study, the feasibility of using visible and near-infrared spectroscopy combined with deep learning to estimate LNC in cotton leaves was explored. The samples were collected from cotton’s whole growth cycle, and the spectra were from different measurement environments. The random frog (RF), weighted partial least squares regression (WPLS), and saliency map were used for characteristic wavelength selection. Qualitative models (partial least squares discriminant analysis (PLS-DA), support vector machine for classification (SVC), convolutional neural network classification (CNNC) and quantitative models (partial least squares regression (PLSR), support vector machine for regression (SVR), convolutional neural network regression (CNNR)) were established based on the full spectra and characteristic wavelengths. Satisfactory results were obtained by models based on CNN. The classification accuracy of leaves in three different LNC ranges was up to 83.34%, and the root mean square error of prediction (RMSEP) of quantitative prediction models of cotton leaves was as low as 3.36. In addition, the identification of cotton leaves based on the predicted LNC also achieved good results. These results indicated that the nitrogen content of cotton leaves could be effectively detected by deep learning and visible and near-infrared spectroscopy, which has great potential for real-world application.

## 1 Introduction

Cotton (*Gossypium* spp), as one of the important economic crops in the world, is widely used in the textile industry because of its excellent natural properties. Nitrogen is an essential plant macronutrient, taking an important part in crop photosynthesis, which provides necessary nutritional support for the growth and development of crops (Ma et al., 2022). Observations have shown that nitrogen fertilization has an important effect on cotton yield. Rational nitrogen fertilization is beneficial to increase cotton yield, while both deficit and excessive nitrogen fertilization have a negative impact on cotton growth and development (Liu et al., 2010; Gospodinova and Panayotova, 2019). Gospodinova and Panayotova (2019) summarized the research on the effects of mineral fertilization on cotton yield and concluded that nitrogen should be applied at different development stages as needed. Optimizing the nitrogen fertilizer application scheme is conducive to improving nitrogen utilization efficiency and cotton yield. Knowing the nutritional status of cotton is the prerequisite to realizing on-demand nitrogen application. Therefore, rapid and accurate evaluation and detection of cotton nitrogen is of great significance for monitoring plant nutrition status, as well as making fertilization decisions.

Leaf nitrogen concentration (LNC), a critical indicator of nitrogen nutrient status, is widely used in crop nutrient status evaluation (Wan et al., 2022). A study conducted by Kergoat et al. (2008) has shown that LNC is an essential factor affecting canopy light utilization efficiency and photosynthetic rate. Generally, LNC is determined by destructive analysis methods, such as the Kjeldahl-digestion method. Although the destructive approaches are objective, they have disadvantages such as being time-consuming, labor-intensive, high cost and strong destructiveness. It is also difficult to meet the actual needs of rapid and real-time detection and diagnosis of LNC in a wide range. In recent years, non-destructive techniques, such as visible and near-infrared (VNIR) spectroscopy (Mishra et al., 2021) and multi-spectral and hyperspectral imaging (Tahmasbian et al., 2021; Guo et al., 2022), have been developed to detect crop nutrition status. Multi-spectral and hyperspectral images usually carry more information than spectra data. However, the acquisition of spectral images generally requires expensive and bulky sensors, the amount of data is enormous, and there is more information redundancy, which requires more storage space and tedious data processing. Multi-spectral and hyperspectral imaging are not economically feasible when many samples need to be examined and evaluated. VNIR allows rapid acquisition of spectral information related to samples’ physiological state and internal components at a relatively low cost. In the past few years, VNIR has attracted extensive attention and has been used in qualitative and quantitative research in plants (Zhang et al., 2020a; Xia et al., 2021; Luo et al., 2022).

For the studies aiming at employing VNIR for nitrogen detection, Mishra (Mishra et al., 2021) et al. demonstrated the feasibility of using VNIR to quantitatively predict the nitrogen and potassium concentration in bell pepper leaves. The results showed that VNIR allowed accurate prediction of nitrogen with an RMSEP of 0.44%. Sun et al. (2013) used VNIR to identify the fertilized nitrogen level of lettuce leaves and achieved a high classification accuracy of 100%. Zhang et al. (2022a) explored the performance of using spectra of different ranges to estimate nitrogen content in cotton leaves and obtained a R^2^_c_ = 0.794~ 0.909 and R^2^_P_ = 0.774 ~ 0.899. Relationships between cotton leaf spectra curves (380-700 nm, 700-1300 nm, and 1300-2500 nm) and nitrogen content contributed to satisfactory predictions for nitrogen content detection. There are indeed many researches on the detection of LNC (Sun et al., 2013; Wang et al., 2018; Gao et al., 2022; Pourdarbani et al., 2022; Tang et al., 2022; Zhang et al., 2022b). Although the studies focusing on the LNC classification achieved good results (Sun et al., 2013; Wang et al., 2018; Pourdarbani et al., 2022), the samples in these studies were classified according to different nitrogen fertilization levels or different nitrogen fertilization days. It should be noted that there is a large difference between the fertilization of nitrogen and its actual uptake for the plant. Therefore, the adaptability of the classification models according to the nitrogen fertilization division is greatly limited by the uncertainty of the actual LNC. What’s more, in practice, it is always hard to get accurate fertilization data and estimate the fertilization condition. In addition, the studies focusing on LNC prediction are mainly for a specific cultivar or a specific spectral data collection environment (Rotbart et al., 2013), which may limit the scope of the applicability of the established models.

Deep learning is a method that simulates the human brain for analysis and learning. It forms abstract features to represent the data distribution. Deep learning has the advantages of strong self-learning and feature-extraction ability and great capability of processing spectra data (Xiao et al., 2020). In recent years, deep learning has been applied to conduct various tasks in spectral and image data processing (Steinbrener et al., 2019; Zhou et al., 2019). Convolutional neural network (CNN) is one of the typical deep learning models. CNN has been proven effective in processing spectra data and establishing classification and regression models for various agricultural tasks (Zhang et al., 2020b; Zhang et al., 2020c; Gai et al., 2022).

The objective of the present study was to explore the feasibility of qualitative diagnosis and quantitative detection of LNC based on VNIR combined with deep learning. The goals include (1) exploring the laws of the spectra of leaves with different LNC; (2) classifying nitrogen levels according to the measured LNC; (3) detecting LNC for two cotton cultivars under the condition that the spectra were collected in different measurement environments. The specific content includes (1) extracting characteristic wavelengths by random frog (RF), weighted partial least squares regression (WPLS), and saliency map for qualitative discrimination and quantitative detection tasks, respectively; (2) building partial least squares discriminant analysis (PLS-DA), support vector machine for classification (SVC), and convolutional neural network classification (CNNC) models based on full spectra and characteristic wavelengths to identify cotton leaves with different LNC qualitatively; (3) developing partial least squares regression (PLSR), support vector machine for regression (SVR), convolutional neural network regression (CNNR) models to quantitatively detect LNC in cotton leaves.

## 2 Materials and methods

### 2.1 Sample preparation

Cotton was planted in an experimental field at the Hangzhou Raw Seed Growing Farm (30°22’58.85” N, 119°56’7.80” E), Hangzhou, Zhejiang province, China. Cotton cultivars Lumianyan 24 (LMY24) and Xinluzao 53 (XLZ53) were sampled in this experiment. Thirty-six experimental plots of 4×2 m were used with six nitrogen rates (0, 120, 240, 360, 480, 278 kg/hm^2^). Each nitrogen level was set with three replicates. Leaf sampling was conducted during the whole growth stage. Leaves at different leaf positions were selected from the experimental plots. Finally, a total of 1400 leaves were acquired. It is worth mentioning that the spectra of 648 leaves were collected in the laboratory, and the spectra of the remaining leaves were collected in the field. For the samples measured in the laboratory, the leaves were cut, placed in the black bags and stored in a cooler with a temperature of about 4°C. These samples were transported to the laboratory immediately. The time of transit was within one hour.

### 2.2 Spectra acquisition

**Figure 1:**
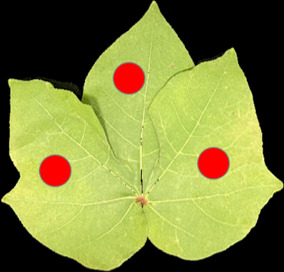
The regions of spectra acquisition for cotton leaves.

Leaf spectra acquisition was conducted by a spectroradiometer (Fieldspec4, Analytical Spectral Devices - ASD, Boulder, CO, USA) system. This spectroradiometer consists of a leaf clip, which provides a light source. During the measurement, the leaves were clamped up for spectra acquisition. Three different positions of each leaf were measured, and five scans were conducted for each measurement. The spectra of five scans were averaged as the spectra of the leaf region, and the average spectra of three leaf regions were taken as the spectra of each leaf. The regions of spectra acquisition for each leaf is shown in **Figure 1**. The collected spectra cover the visible and near-infrared region (400 ~ 1000 nm) and the short-wave near-infrared region (1000 ~ 2500 nm), and the spectral resolution are 3nm and 8nm, respectively. Considering the noise in the beginning, spectra between 430-2500 nm were used in this study.

### 2.3 Measurements of leave nitrogen concentration

**Figure 2:**
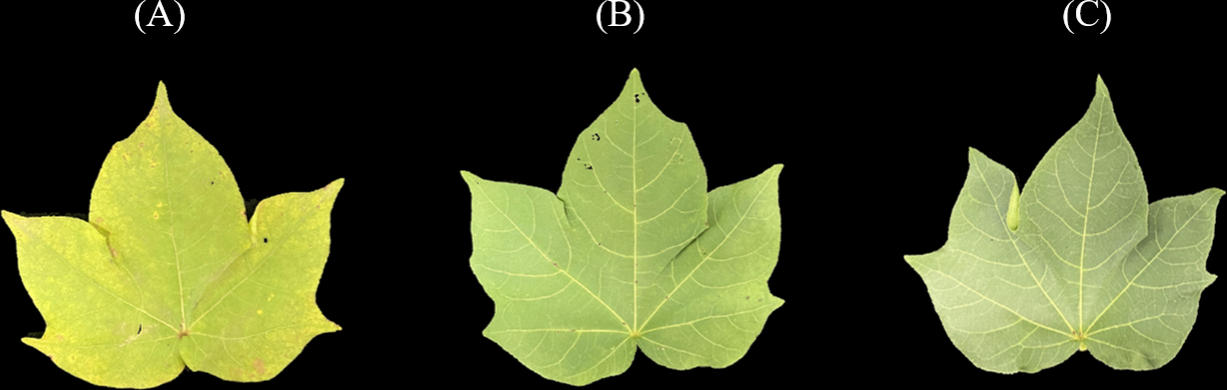
Cotton leaves with different LNC levels: **(A)** low-level LNC, **(B)** medium-level LNC, **(C)** high-level LNC.

After finishing the spectra collection, the leaves were placed in an oven, dried at 105°C for half an hour, and then cooled to 80°C until the sample weight recorded a constant weight. Then, the dried leaves were ground into a fine powder and sieved through a 40-mesh. A uniform dry leaf sample of fixed mass was taken, and the nitrogen concentration was determined by the Kjeldahl method after acid digestion (Kjeldahl, 1883). According to the measured LNC (mg/g), cotton leaf samples were divided into three categories: low-level LNC, medium-level LNC, and high-level LNC. The detailed statistical information on sample composition is presented in **Table 1**. Cotton leaves with different LNC levels are shown in **Figure 2**. It can be seen that the leave with high-level LNC has a deeper green color.

**Table 1:** Statistical information of composition of the cotton leaves.

LNC level	total number of samples	Range of LNC (g/kg)	Mean (g/kg)	Standard Deviation	number of samples incal/val/pre set
Low	230	14.99-25.00	21.73	2.37	138/46/46
Medium	601	25.02-34.96	30.09	2.84	360/120/121
High	569	35.02-52.46	40.19	3.60	341/114/114

cal/val/pre set means the calibration set, the validation set and the prediction set, respectively.

### 2.4 Data analysis methods

#### 2.4.1 Convolutional neural network

**Figure 3:**
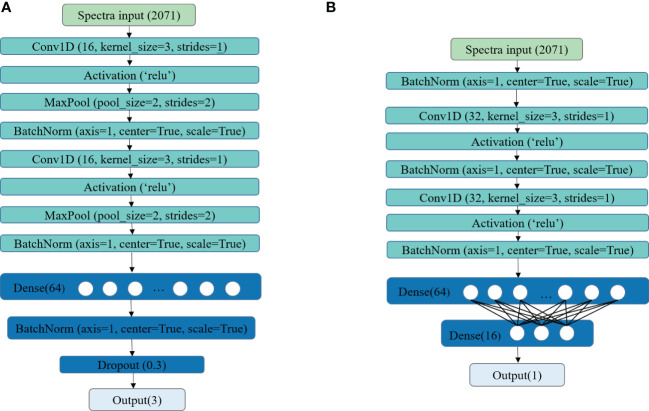
The CNN structure for classification model **(A)** and regression model **(B)**.

In this study, two self-developed CNN architectures were applied for building classification and regression models, and their structures are shown in **Figures 3A, B**, respectively. For the classification task, two convolution layers were set, both followed by a max pooling layer and a batch normalization layer. The number of filters, kernel size, and strides of the two convolution layers were both set as 16, 3, and 1, respectively. The rectified linear unit (ReLU) was used as the activation for computing the outputs of the convolutional layers. The max pooling layer served as down-sampling and dimensionality reduction to form the features of the next layer. Then, a fully connected network with 64 neurons was added, followed by a batch normalization layer. The dropout layer was used to avoid overfitting. The fully connected layer at the end was used for output. For the regression task, two batch normalization layers followed by convolution layers were employed. The number of filters, kernel size, and strides of the two convolution layers were both set as 32, 3, and 1, respectively. Same as the proposed CNN for classification, the rectified linear unit (ReLU) was used as the activation. A batch normalization layer was added before the features were outputting to the fully connected layer. In the end, two fully connected layers with 64 and 16 neurons were used for building non-linear regression models to predict the LNC of different leaves. The fully connected layer at the end was used for output.

For the training phase, the Softmax cross-entropy loss function combined with stochastic gradient descent (SGD) optimizer was applied to train the CNN developed for the classification task. The L1 loss function and the adaptive moment estimation (Adam) optimizer were used for the regression task. The detailed information about SGD optimizer and Adam optimizer could be found on the website https://pytorch.org/docs/stable/optim.html . For both training tasks, the batch size was set as 64, and a scheduled learning rate was used. In the beginning, the learning rate was set to 0.05. The learning rate was reduced ten times after every 200 epochs. According to this rule, the training phase was terminated when the loss was stable.

#### 2.4.2 Conventional models

PLS-DA and SVC models were established to classify cotton leaves with different LNC. PLS-DA is a linear discriminant algorithm developed from PLSR (Yuan et al., 2021). PLS-DA algorithm can effectively extract the variables helpful for classification and realize data recognition. PLS-DA can deal with irreversible matrices and select the number of latent variables so that the model achieves the best balance between underfitting and overfitting (Li et al., 2021). SVC is a pattern recognition algorithm based on a support vector machine (SVM) for classification. It achieves the classification goal by exploring the hyperplane that maximizes the distance between different classes (Xiao et al., 2020). In this study, the radial basis function (RBF) was used as the kernel function. The regularization parameter c and kernel function parameter g were determined through a grid-search procedure. The search range of c and g were both assigned as 2^-8^ to 2^8^. PLSR and SVR models were used to establish the quantitative analysis model of LNC. Detailed information on PLS and SVR details can be found in our previous article (Xiao et al., 2022). For both qualitative and quantitative analysis models, five-fold cross-validation was adopted.

#### 2.4.3 Wavelengths selection

Hyperspectral data contains massive amounts of information, which also exists information redundancy, collinearity, and noise that are not conducive to data processing. To make effective use of data, characteristic wavelengths extraction is a common strategy. In this study, RF, WPLS and saliency map were used to extract characteristic wavelengths. For the RF algorithm, based on the idea of inversible-jump Markov Monte Carlo, PLS-DA and PLSR are selected as modeling methods for classification and regression, respectively. Models are established by constantly updating the subset of variables according to the defined criteria. The frequency of each variable selected in the modeling subset is calculated after reaching the number of iterations (Yun et al., 2013; Sun et al., 2021). The top 40 wavelengths with the highest frequency were selected as the characteristic wavelengths. When using WPLS for wavelength selection, a PLS regression model is first established, and each variable’s regression coefficient was calculated. The wavelengths with the larger absolute value of the regression coefficient at the crest and trough were selected (Mehmood et al., 2012). Saliency map is a popular method for computing the contribution of each variable to the model performance. In this study, for classification tasks, CNNC model was first established and calculated the saliency based on the method proposed in Feng’s study (Feng et al., 2021). Similarly, as for regression tasks, CNNR model was first established and saliency map was applied following the way in our previous study (Xiao et al., 2022). The first 40 critical wavelengths with the highest frequency for both tasks were selected as the characteristic wavelengths.

#### 2.4.4 Software and model evaluation

For model establishment, PLS-DA and PLSR were performed in R2019b (The MathWorks, Natick, MA, USA). SVC, and SVR were conducted in the scikit-learn 0.23.1 (Anaconda, Austin, TX, USA) using python 3.1. The CNN models were conducted in MXNet 1.4.0 (MXNetAmazon, Seattle, WA, USA). For feature selection, RF was performed in R2019b (The MathWorks, Natick, MA, USA). WPLS was carried out in the Unscrambler X 10.1 (Camo AS, Oslo, Norway). Saliency map was conducted in MXNet1.4.0 (MXNetAmazon, Seattle, WA, USA).

It is critical to evaluate the model performance with appropriate indicators. Classification accuracy is used for assessing the qualitative analysis models. Classification accuracy is calculated as the ratio of correctly classified samples to the total number of samples. The closer it is to 100%, the better the model’s performance. The coefficients of determination (R^2^) and root mean square error (RMSE) of calibration, validation, and prediction set were applied to assess the performance of quantitative analysis models. The closer R^2^ of the model is to 1, the closer RMSE is to 0, indicating that the model performance is more satisfactory.

## 3 Results

### 3.1 Spectra features

**Figure 4:**
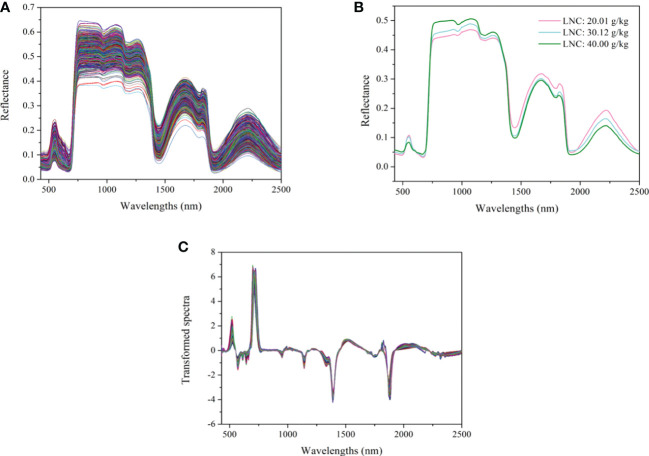
Spectra of cotton leaves: **(A)** the spectra of all the cotton leaves, **(B)** the spectra of leaves with different LNC, **(C)** the spectra transformed by FD +SNV.

The spectra of all the cotton leaves and leaves with different LNC are shown in **Figures 4A, B**. As shown in **Figure 4A**, the spectra of all the leaves present a consistent change tendency. Four peaks (550, 1650, 1820, and 2225 nm) and three valleys (670, 1432, and 1950 nm) were observed. The analysis of the chemical bonds which may be assigned to the peaks and valleys can be found in our previous study (Xiao et al., 2022). **Figure 4B** presents the spectral curves of cotton leaves with different LNC. It can be seen that the reflectance of leaves with high LNC in the range of 430 ~ 520 nm is slightly higher than that of leaves with low LNC. There is a slight increase in the reflectance between 520 ~ 610 nm, and the reflectance of leaves with low LNC becomes higher. After 700 nm, the reflectance curves increase sharply and form a high reflectivity plateau between 775 and 1300 nm, between which the reflectance of leaves with high LNC is larger. The variation trend of the reflectance with LNC between the range of 1400 ~ 1900 nm and 2000 ~ 2500 nm is the opposite from that in the range of 775-1300 nm. The variation of reflectance in different spectral intervals makes it possible to identify leaves with different LNC content. As the results demonstrated in our previous study (Xiao et al., 2022), the model based on the spectra processed by first derivative (FD) and standard normal variate transformation (SNV) demonstrated great generalization ability. Therefore, the method of FD+SNV was used to preprocess the spectra and the processed spectra were used for subsequent modelling. The transformed curves are shown in **Figure 4C**.

### 3.2 Wavelengths selection

Hyperspectral data contains amounts of information such as redundancy, collinearity, background, and other information unrelated to LNC detection. The irrelevant information will significantly increase the burden of data processing, affect the analysis and extraction of effective data, and directly affect the model’s performance. Therefore, in this study, RF, WPLS, and saliency map were used to select the characteristic wavelengths.

The optimal wavelengths selected by different methods for regression models and classification models are shown in **Tables 2** and **3**, respectively. It can be seen that the number and the location of the selected wavelengths on spectral curves varied from different methods. For the wavelengths selected for the classification model, compared with full spectra, the number of variables chosen by RF, WPLS, and saliency map was reduced by 98.07%, 98.02%, and 98.07%, respectively. For the wavelengths selected for the regression model, the number of variables selected by RF, WPLS, and saliency map was reduced by 98.07%, 97.73%, and 98.07%, respectively. Obviously, wavelengths selection significantly reduces data computation and alleviates the model dependence on high-performance computing instruments, which will contribute to the popularization and application of the model.

**Table 2:** The optimal wavelengths selected by RF, WPLS, and saliency map for regression models.

Method	Number	Optimal wavelength (nm)
RF	40	594, 602, 776, 1104, 1109, 1139, 1144, 1196, 1232, 1242, 1274, 1472, 1473, 1490, 1516, 1534, 1568, 1597, 1600, 1604, 1615, 1619, 1620, 1663, 1671, 1741, 1742, 1797, 1897, 1953, 1958, 1963, 1989, 2012, 2047, 2103, 2106, 2134, 2135, 2211
WPLS	47	436, 464, 535, 555, 565, 573, 594, 607, 638, 655, 688, 699, 755, 760, 845, 936, 957, 973, 1179, 1357, 1387, 1409, 1490, 1673, 1690, 1711, 1720, 1746, 1773, 1785, 1810, 1822, 1876, 1894, 2046, 2066, 2154, 2188, 2236, 2254, 2273, 2317, 2335, 2355, 2459, 2478, 2488
Saliency map	40	461, 551, 552, 553, 554, 1177, 1178, 1179, 1670, 1671, 1672, 1673, 1674, 1675, 1676, 1677, 1678, 1679, 1680, 1681, 1682, 1683, 1684, 1699, 1700, 1701, 1702, 1710, 1783, 1784, 1785, 2151, 2152, 2153, 2227, 2228, 2229, 2230, 2458, 2459

**Table 3:** The optimal wavelengths selected by RF, WPLS, and saliency map for classification models.

Method	Number	Optimal wavelength (nm)
RF	40	594, 595, 776, 1139, 1195, 1232, 1233, 1275, 1471, 1472, 1490, 1533, 1534, 1568, 1597, 1600, 1604, 1615, 1616, 1619, 1620, 1621, 1648, 1663, 1671, 1738, 1741, 1745, 1746, 1798, 1953, 1958, 2021, 2044, 2047, 2103, 2106, 2134, 2135, 2211
WPLS	41	432, 437, 444, 464, 525, 556, 564, 571, 595, 607, 638, 678, 688, 698, 755, 936, 963, 997, 1074, 1176, 1355, 1370, 1387, 1672, 1690, 1711, 1720, 1746, 1785, 1810, 1822, 1876, 1897, 2155, 2273, 2324, 2335, 2355, 2479, 2487, 2490
Saliency map	40	957, 976, 977, 978, 1002, 1193, 1670, 1671, 1672, 1674, 1680, 1682, 1690, 1700, 1702, 1703, 1704, 1718, 1720, 1722, 1723, 1818, 2131, 2133, 2137, 2147, 2149, 2152, 2155, 2156, 2157, 2160, 2228, 2230, 2231, 2232, 2234, 2235, 2236, 2333

**Figure 5:**
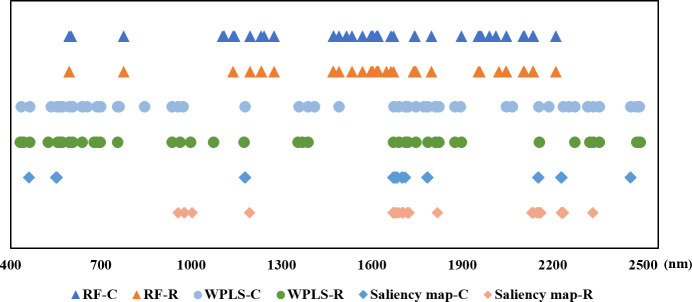
The position of the optimal wavelengths selected for classification and regression models (-C means for classification tasks, -R means for regression tasks).

The position of the selected wavelengths in the spectral curve is displayed in **Figure 5**. For specific wavelength selection methods, the position of characteristic wavelength selected for classification and regression is largely coincidental. It indicated that the characteristic wavelengths related to nitrogen detection selected by wavelength selection method were consistent even in the tasks with different purposes. The specific number of selected variables might be related to the calculation protocol of wavelength selection method. Although there are differences in the number and location of wavelengths selected by RF, WPLS, and saliency map, some bands were chosen as the optimal wavelengths for LNC detection by more than one method, such as the bands around 554 nm, 595 nm, 1179nm, 1490 nm, 1671nm, 1673 nm, 1746 nm, 2046 nm, 2154 nm, 2230 nm, and 2459 nm. These bands are likely to have a strong correlation with nitrogen detection. Among the wavelengths selected by more than one algorithm, the bands in visible range was related with the color of the leaves (Malacara, 2011). The spectral response near 1490 nm was associated with N-H amide with N-R group, which can be connected with protein content (Salzer, 2008). The reflectance around 1673 nm and 1746 nm were associated with C-H methyl (Salzer, 2008). The bands around 2046 nm was due to symmetrical NH stretching and amide II (Salzer, 2008).

### 3.3 Classification models

#### 3.3.1 Classification models using full spectra

**Figure 6:**
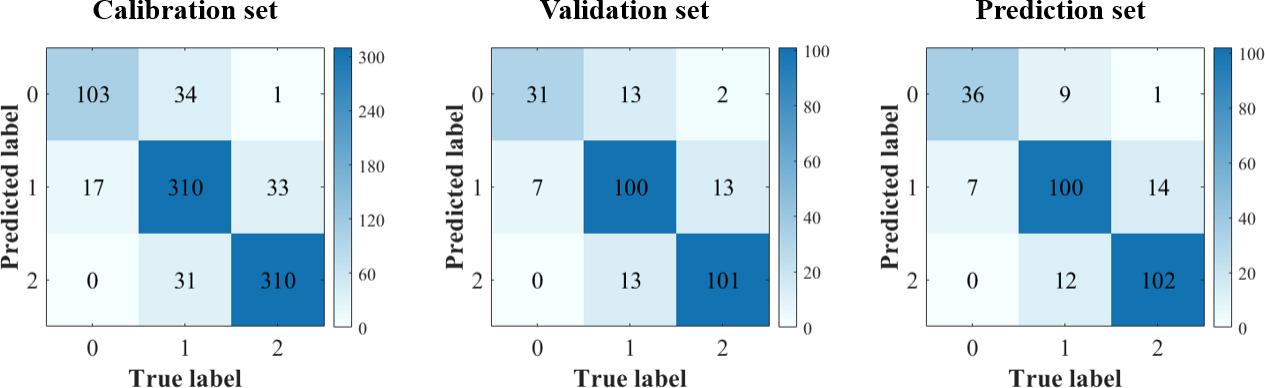
Confusion matrix of CNN model using full spectra. (Notes: Number 0, 1, 2 means leaf samples with low-level LNC, medium-level LNC and high-level LNC, respectively.).

In this study, PLS-DA, SVC, and CNNC models were built using full spectra. The results are shown in **Table 4**. All the models obtained decent results, with the accuracy of the prediction set exceeding 82%. Compared with PLS-DA and SVC models, the CNNC model achieved a more satisfactory result. The accuracy of the prediction set was 84.70%, which illustrates the good performance of CNNC model. The confusion matrix for all datasets of the CNNC model is displayed in **Figure 6**. In three data sets, about 19-28% of leaf samples with low LNC were easily confused with leaves with medium LNC. Leaves with high LNC were easily confused with those with medium LNC, and the proportion of misclassified leaves was 8%-11%. Overall, leaves with high LNC and leaves with low LNC can be accurately separated, as only one or two leaves with low LNC were misclassified as high LNC, and none samples with high LNC were distinguished as low LNC.

**Table 4:** The results of the classification models based on full spectra.

Model	Accuracy		
	Calibration set	Validation set	Prediction set
PLS-DA	88.56%	77.14%	82.56%
SVC	85.10%	79.29%	83.99%
CNN	86.17%	82.86%	84.70%

#### 3.3.2 Classification models using optimal wavelengths

**Table 5** demonstrates the classification results of the PLS-DA, SVC, and CNNC models based on the optimal wavelengths selected by RF, WPLS, and saliency map. The overall performance of the models based on wavelengths selected by RF was superior to the performance of the models constructed on the wavelengths selected by WPLS and saliency map, as the accuracy for the prediction set was slightly higher. For the models based on the optimal wavelengths selected by RF and WPLS, the performance of the CNNC model was superior to that of the PLS-DA and SVC model, accomplishing an accuracy of 84.34% and 83.27% for the prediction set. Regarding the models based on the wavelengths selected by saliency map, SVC performed better than the PLS-DA and CNNC model, with the accuracy of the prediction set reaching 79.36%. Although the CNNC model based on full spectra achieved the best classification accuracy of 84.70% for the prediction set, the CNNC model based on the optimal wavelengths chosen by RF obtained quite similar results. Considering the number of variables used in modeling, the results of CNNC models constructed on the optimal wavelengths selected by RF were reasonably acceptable, which realized comparable performance with the model based on full spectra with less computation.

**Table 5:** The results of the classification models based on optimal wavelengths.

Data type	Model	Accuracy		
		Calibration set	Validation set	Prediction set
RF	PLS-DA	80.93%	75.71%	79.00%
	SVC	88.08%	80.71%	83.99%
	CNN	86.53%	79.64%	84.34%
WPLS	PLS-DA	78.90%	77.86%	77.22%
	SVC	84.03%	80.36%	82.92%
	CNN	82.84%	79.29%	83.27%
Saliency map	PLS-DA	74.37%	71.89%	74.29%
	SVC	84.51%	78.21%	79.36%
	CNN	83.08%	77.50%	77.58%

### 3.4 Regression models

#### 3.4.1 Regression models using full spectra

**Figure 7:**
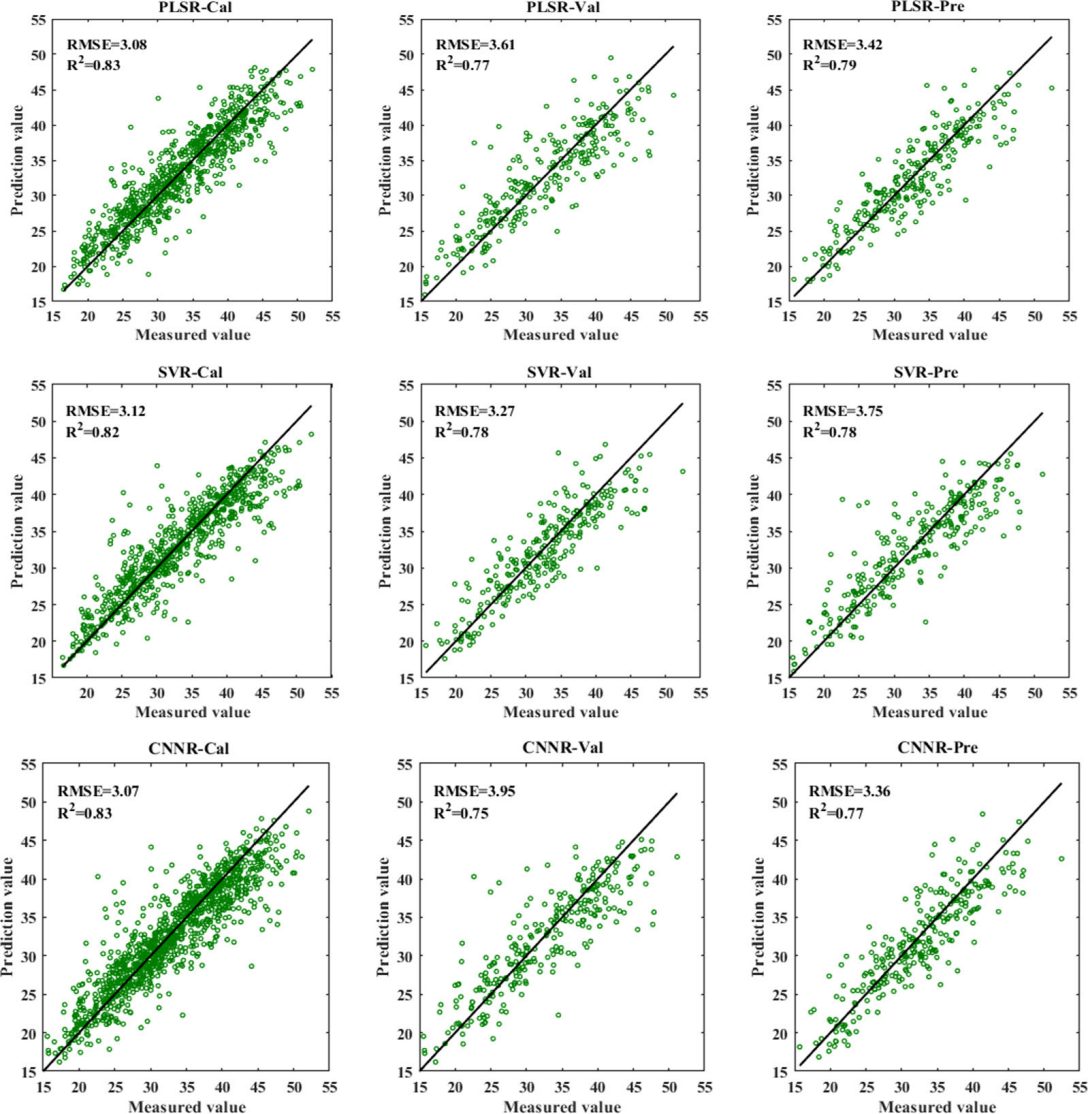
The results of PLSR, SVR, CNNR models based on full spectra for LNC detection.

**Figure 7** shows the results of different regression models using full spectra for the nitrogen detection of cotton leaves. All the models obtained satisfactory performance, with R^2^_c_ (coefficients of determination of calibration set), R^2^_v_ (coefficients of determination of validation set), and R^2^_p_ (coefficients of determination of prediction set) all exceeding 0.75. Compared with PLSR and SVR models, the CNNR model performed slightly better, achieving the smallest RMSE for the prediction set. These results indicated that VNIR combined with the CNNR model was conducive to effectively characterizing the LNC of cotton leaves.

#### 3.4.2 Regression models using optimal wavelengths

Different regression models were constructed based on the optimal wavelengths for LNC estimation. The results are shown in **Table 6**. It can be found that the performance of the model established using the optimal wavelengths selected by different methods showed a difference. The models built on the optimal wavelengths selected by WPLS performed slightly better than the models based on the optimal wavelengths selected by RF and saliency map, with higher R^2^_P_ and lower RMSEP. In addition, it can be found that the overall performance of CNNR models was better than that of PLS and SVR models. All the CNNR model achieved good results, with R^2^_c_, R^2^_v_, and R^2^_p_ were over 0.779, 0.724, and 0.711, indicating the robustness of the CNNR model based on optimal wavelengths. Specifically, the CNNR model based on the wavelengths chosen by WPLS obtained the best result. The R^2^_p_ and RMSEP were 0.766 and 3.389, respectively. Besides, a comparison was made between the models based on full spectra and those using the selected optimal wavelengths. Overall, the models established on the chosen variables performed less well than those based on full spectra. The performance of the CNNR model based on optimal wavelengths selected by WPLS was quite close to that based on full spectra. To some extent, the reduced computation compensates for the slight performance deficit, indicating that the CNNR model equipped with optimal wavelength selection methods is effective for cotton LNC estimation.

**Table 6:** The results of the regression models based on optimal wavelengths.

Data Type	Model	Calibration set		Validation set		Prediction set	
		R^2^_C_	RMSEC	R^2^_V_	RMSEV	R^2^_P_	RMSEP
RF	PLSR	0.76	3.67	0.75	3.95	0.74	3.59
	SVR	0.80	3.30	0.73	3.62	0.73	4.12
	CNNR	0.82	3.19	0.74	4.02	0.75	3.53
WPLS	PLSR	0.78	3.52	0.76	3.93	0.76	3.42
	SVR	0.80	3.37	0.76	3.42	0.77	3.83
	CNNR	0.79	3.38	0.76	3.91	0.77	3.39
Saliency map	PLSR	0.66	4.36	0.60	2.02	0.67	4.03
	SVR	0.81	3.27	0.71	3.77	0.72	4.17
	CNNR	0.78	3.50	0.72	4.17	0.71	3.76

#### 3.4.3 Identification of leaf nitrogen status based on the predicted LNC

The predicted LNC values of all samples were calculated by regression model and then classified according to the rules mentioned in section 2.3. Then, the identification accuracy was calculated by comparing the categories corresponding to the predicted and actual values to evaluate the model’s effectiveness. The results are shown in **Table 7**. It can be seen that the identification of cotton samples based on the predicted value could achieve good results, similar to that of the results based on classification models, which in turn reflects the effectiveness of the regression model.

**Table 7:** The identification results of cotton leaves based on the predicted LNC.

Data type	Model	Accuracy		
		Calibration set	Validation set	Prediction set
Full spectra	PLSR	83.81%	80.71%	85.00%
	SVR	85.71%	83.21%	79.29%
	CNNR	84.17%	78.21%	83.21%
RF	PLSR	81.43%	76.79%	83.21%
	SVR	85.00%	81.79%	80.00%
	CNNR	83.57%	77.50%	81.43%
WPLS	PLSR	81.07%	78.57%	81.79%
	SVR	82.50%	81.79%	78.21%
	CNNR	85.12%	78.93%	81.07%
Saliency map	PLSR	75.60%	64.64%	73.93%
	SVR	84.40%	78.21%	78.21%
	CNNR	81.90%	76.07%	77.14%

## 4 Discussion

Visible and near-infrared spectral techniques combined with deep learning can be used for nitrogen-level estimation. Some previous studies addressing the nitrogen-level classification of plant leaves were discussed and compared with our results with other spectral imaging works. It must be noted a rough comparison is not rigorous as the papers relate to different plants, techniques, and datasets. Pourdarbani et al. (2022) investigated the feasibility of using hyperspectral imaging to detect excess nitrogen content in tomato plants. Artificial neural networks and the particle swarm optimization algorithm were proposed and achieved a satisfactory classification accuracy of 92.6% for leaves at different nitrogen levels. The leaves in this work (Pourdarbani et al., 2022) were classified according to different days of nitrogen application. Sun et al. (2013) used VNIR to identify the nitrogen level of lettuce leaves. Adaptive boosting was applied with K nearest neighbor and SVM, which could achieve a high classification accuracy of 100%. The samples in this study were divided according to the fertilized nitrogen level. Wang et al. (2018) employed hyperspectral imaging to discriminate nitrogen fertilizer levels of the tea plant. The leaves from three nitrogen fertilizer levels were sampled, and up to 100% accuracy was achieved by the SVM model based on spectral data and textural data. The excellent performance might benefit from the texture information provided by the image. Although the methods mentioned above achieved good results, the samples in these studies were classified according to different nitrogen fertilization levels or different nitrogen fertilization days (Sun et al., 2013; Wang et al., 2018; Pourdarbani et al., 2022). There is a large difference between the fertilization of nitrogen and its actual uptake for the plant. Therefore, the adaptability of the classification models according to the nitrogen fertilization division is greatly limited by the uncertainty of the actual LNC. Thus, in this study, the chemical analysis for nitrogen measurement was conducted, and the leaves were divided into three categories according to the true LNC value of three ranges. Among similar studies which also measured actual LNC, Nativ et al. (Rotbart et al., 2013) used VNIR to estimate the nitrogen concentration in olive leaves. The leaves were divided into three groups according to the measured nitrogen content, and an overall accuracy of 83% was obtained. Du et al. (2016) explored the feasibility of using hyperspectral LiDAR to detect nitrogen content in rice leaves. The accuracy of 83% was obtained when 32 wavelengths were considered. The results in above studies (Rotbart et al., 2013) (Du et al., 2016) are slightly lower than the accuracy of 84.342% achieved by RF-CNN model in this study. It can be observed that although a perfect classification is not achieved, the method used in this study has a relatively higher accuracy of 84.342% in the best case. The performance is quite close to and even higher than the result obtained by other existing methods, which demonstrates that it is feasible to classify cotton leaves with different LNC by VNIR and deep learning algorithm.

Regarding the regression task, in a similar study on the LNC prediction of cotton leaves, Zhang et al. (2022a) explored the potential of using spectra of different ranges to estimate nitrogen content in cotton leaves, and obtained a R^2^_c_ = 0.794~ 0.909 and R^2^_P_ = 0.774 ~ 0.899. The prediction results of the best model are better than those in this research. The possible reason was that the samples used in this study (Zhang et al., 2022) were acquired at the flower and boll stage of cotton, which only covered two growing stages. The leaves used in our study cover the whole growing stage. Different thicknesses and textures of leaf samples would also cause spectral differences, which may affect the accuracy of nitrogen detection. Besides, the spectra in this paper were collected under two environmental conditions, covering the laboratory environment and the field environment. The difference in the measurement environment would also lead to the difference in the spectra. When the measurement was conducted in the field, there were more interference factors, which was also the reason for the relatively less satisfactory results. However, in practice, due to the diversity of application scenarios and the need for nutrient monitoring over the whole growth cycle of plants, it is critical to develop the models presented in this paper to enhance their applicability.

Besides, deep learning with VNIR performed well in estimating LNC in plant leaves. **Table 6** and **Figure 7** show that CNNR outperformed PLSR and SVR models, achieving a relatively lower RMSE for the prediction set. The study demonstrated that the CNN model established for regression tasks could achieve good results, which previous studies have confirmed. Weng et al. (2022) combined CNNR and visible and near-infrared reflectance spectroscopy to determine the behenic acid in edible vegetable oils, with R^2^_P_ = 0.8843 and RMSEP = 0.1182, outperforming PLSR and SVR model. Wu et al. (2022) applied CNNR and Raman spectroscopy to identify the amount of olive oil in a corn-olive oil blend, with R^2^_P_ = 0.9908 and RMSEP = 0.7183. In addition, one-dimension deep learning regression models based on spectral data are performed well in soluble solid content estimation in cherry tomato (Xiang et al., 2022) and oil content prediction of single maize kernel (Zhang et al., 2022c). Hence, the spectral analysis model developed by CNN can be expected to provide a simple, rapid, and accurate analysis of LNC in cotton leaves.

## 5 Conclusion

In this study, visible and near-infrared spectroscopy combined with deep learning was used to detect LNC in cotton leaves qualitatively and quantitatively. RF, WPLS, and saliency map were used to extract characteristic wavelengths, classification models (PLS-DA, SVC, CNNC) and regression models (PLSR, SVR, CNNR) were established based on full spectra and characteristic wavelengths, respectively. Overall, the models based on CNN architecture performed better than other models for both classification and regression tasks. For the classification task, CNNC model based on full spectra performed best, with the classification accuracy reaching 84.70%. For the regression task, the performance of CNNR model developed on full spectra was superior, achieving an R^2^_P_ of 0.77 and an RMSEP of 3.36. The good performance of visible and near-infrared spectroscopy assisted by deep learning demonstrated its effectiveness for nitrogen content prediction of cotton leaves. This approach is helpful for farmers to accurately identify the nutritional status of cotton plants in the field and make reasonable fertilization decisions in time.

## Data availability statement

The raw data supporting the conclusions of this article will be made available by the authors, without undue reservation.

## Author contributions

YH, LF, and NW, funding acquisition, conceptualization, and supervision. QX, WT, and NW, data curation and validation. CZ, LF, LZ, ZZ, and PG, formal analysis, and writing, project administration review and editing. CZ and QX, investigation, methodology, and software. JS, resources. QX, NW, and CZ, visualization and writing, original draft. All authors contributed to the article and approved the submitted version.
